# The Treatment Outcome and Radiation-Induced Toxicity for Patients with Head and Neck Carcinoma in the IMRT Era: A Systematic Review with Dosimetric and Clinical Parameters

**DOI:** 10.1155/2013/401261

**Published:** 2013-10-22

**Authors:** Vassilis Kouloulias, Stella Thalassinou, Kalliopi Platoni, Anna Zygogianni, John Kouvaris, Christos Antypas, Efstathios Efstathopoulos, Kelekis Nikolaos

**Affiliations:** ^1^Second Department of Radiology, Radiotherapy Unit, Attikon University Hospital, Medical School, Rimini 1, Xaidari, 12462 Athens, Greece; ^2^First Department of Radiology, Radiotherapy Unit, Aretaieion University Hospital, Medical School, Vas. Sofias 76, 11528 Athens, Greece

## Abstract

A descriptive analysis was made in terms of the related radiation induced acute and late mucositis and xerostomia along with survival and tumor control rates (significance level at 0.016, bonferroni correction), for irradiation in head and neck carcinomas with either 2D Radiation Therapy (2DRT) and 3D conformal (3DCRT) or Intensity Modulated Radiation Therapy (IMRT). The mean score of grade > II xerostomia for IMRT versus 2-3D RT was 0.31 ± 0.23 and 0.56 ± 0.23, respectively (Mann Whitney, *P* < 0.001). The parotid-dose for IMRT versus 2-3D RT was 29.56 ± 5.45 and 50.73 ± 6.79, respectively (Mann Whitney, *P* = 0.016). The reported mean parotid-gland doses were significantly correlated with late xerostomia (spearman test, rho = 0.5013, *P* < 0.001). A trend was noted for the superiority of IMRT concerning the acute oral mucositis. The 3-year overall survival for either IMRT or 2-3DRT was 89.5% and 82.7%, respectively (*P* = 0.026, Kruskal-Wallis test). The mean 3-year locoregional control rate was 83.6% (range: 70–97%) and 74.4 (range: 61–82%), respectively (*P* = 0.025, Kruskal-Wallis). In conclusion, no significant differences in terms of locoregional control, overall survival and acute mucositis could be noted, while late xerostomia is definitely higher in 2-3D RT versus IMRT. Patients with head and neck carcinoma should be referred preferably to IMRT techniques.

## 1. Introduction

 Over the last years, radiotherapy has played a significant role in the treatment of head and neck cancers. 74% of head and neck cancer patients need to undergo either definitive or postoperative radiation therapy [1]. The transition from two-dimensional conventional radiotherapy (2D-RT) to three-dimensional conformal radiotherapy (3D-CRT), in addition to further technological evolutions in the field of radiotherapy, led to the successful clinical implementation of intensity modulated radiation therapy (IMRT) which constitutes an evolution of 3D-CRT [2]. The IMRT has been employed in clinical practice since 1995 resulting in a great specimen of clinical results from patients undergone this specific technique of radiotherapy [3]. The IMRT technique gives the ability to create treatment fields with varying beam intensity by using inverse planning and iterative optimization algorithms [4]. The irradiation beam can be adjusted to the irregularly shaped target volumes with extremely high precision whilst reducing the radiation delivered to the surrounding healthy tissue and critical structures such as spinal cord, brain stem, parotid glands, eyes, optic nerves, chiasma, lacrimal glands, cochlea, and mandible in case of head and neck cancer [5–7]. The ability of delivering lower doses of radiation to normal tissue while maintaining or increasing the dose in the target volume makes IMRT the most appropriate treatment option compared to 2D-RT and 3D-CRT [8–12].

Radiation therapy causes acute and late toxicities that affect various organs and functions. One of the most common acute toxicities that occurs as an injury of the mucosa of the head and neck area due to irradiation is mucositis. In the case of late toxicity, the most common characteristic is xerostomia where the considerable reduction of saliva leads to persistent dryness of mouth, oral discomfort, sore throat, dental decay, difficulty in speech, taste alteration, and impairment of chewing and swallowing functions which can lead to nutritional depletion and weight loss [13–16]. According to the published results, IMRT technique improves the toxicity profiles without compromising the efficacy [9, 12, 17–19]. The reduction of acute and late toxicities [8, 9, 12, 18, 20–22] in conjunction with comparable or superior treatment outcomes [8–10, 20, 21, 23] increases the necessity of IMRT technique for the treatment of head and neck carcinomas.

The objective of this study is to review the already published results and compare the efficacy and toxicity between patients treated with conventional RT techniques (2DRT and 3DCRT) and those treated with IMRT technique for head and neck carcinomas.

## 2. Materials and Methods

The literature was accessed through PubMed and Scopus (March 2000–January 2013), using the terms “radiation therapy,” “head and neck cancer,” “toxicity,” “tumor control,” and “survival.” Additional papers were identified by cross-referencing bibliographies of retrieved articles. Tumor control and survival outcomes for head and neck cancers were collected from 38 studies while outcomes of acute and late toxicity were collected from 33 studies. As far as toxicity is concerned, two of the most common acute and late radiation-induced morbidity were included such as mucositis and xerostomia. The mean parotid-gland dose was also recorded as it contributes to radiation-induced xerostomia. The published results were categorized according to the radiation therapy technique which was used for the treatment of head and neck carcinomas in order to estimate the differences in clinical outcomes. The present review study focused mainly on hypopharyngeal, nasopharyngeal, and oropharyngeal tumors as well as on tumors of the larynx and the oral cavity. Furthermore, clinical outcomes for curative reirradiation were not included in the collected data.

### 2.1. Statistical Analysis

The analysis included a statistical correlation with spearman-rho nonparametric test between either the RT technique (IMRT versus 2-3D RT) or the mean parotid dose and the incidence of late xerostomia. For the analysis of the differences of the doses at the parotids and the mean score of xerostomia stratified by the RT technique, we used the Mann-Whitney test. The potential impact of RT technique to either survival or locoregional control rate was evaluated with the Kruskal-Wallis test. According to the bonferroni correction, the significance level was set at 0.016. Due to the efficient number of data concerning the survival and locoregional control rate of the 3-year-followup, we decided to make the analysis for the 3-year survival and locoregional rate. The statistical analysis was performed with the SPSS version 10 software (Chicago, IL, USA).

## 3. Results

According to the published data, the head and neck primary site was as follows: oropharynx 41%, nasopharynx 37%, oral cavity 6%, larynx/hypopharynx 15%, and other tumor site 1%. Among the studies, 2582 out of 4587 patients (56%) received concurrent chemotherapy. In terms of radiation therapy technique, IMRT was given to 3618 out of 4587 patients (79%) and 2-3D RT was given to 969 out of 4587 patients (21%). Definitive versus post-operative RT was given to 3953 out of 4587 (86%) versus 633 out of 4587 (14%) patients, respectively. 

**Figure 1:**
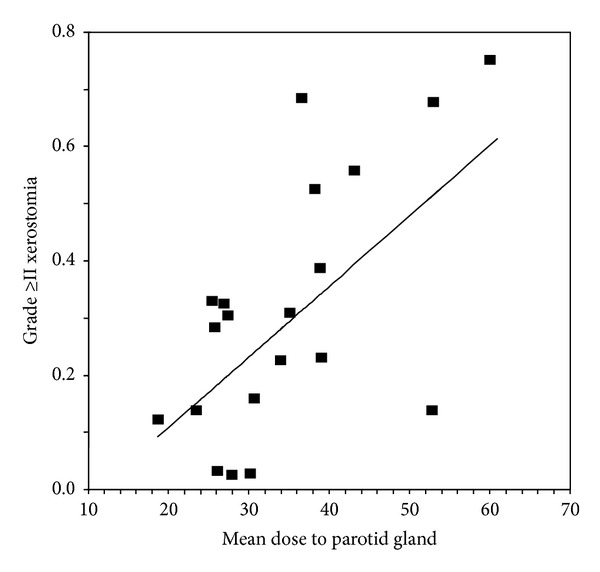
Linear curve estimation for grading ≥ II xerostomia related to the mean dose of parotid gland (rho = 0.5013, *P* < 0.001). The analysis was performed from 20 published trials with relevant available data.

**Figure 2:**
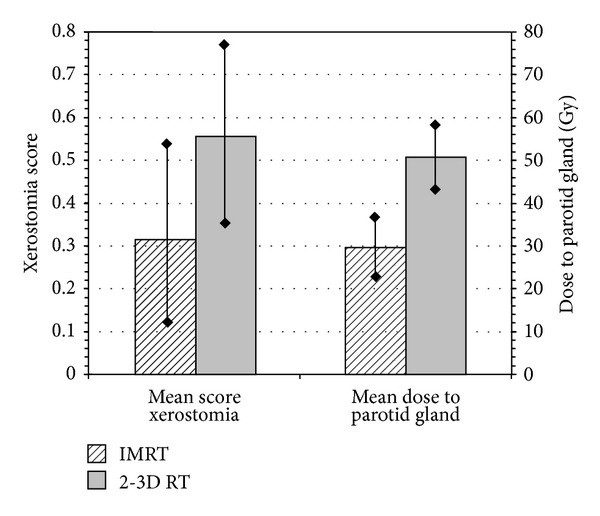
Comparative descriptive analysis of mean dose to parotids and mean xerostomia score stratified by RT technique. A significant difference was noted between the IMRT and conventional technique for both doses to parotids (*P* = 0.016) and xerostomia (*P* < 0.001). The related scale referring to the *y*-axis either right or left is presenting the dose to the parotids and the xerostomia score, respectively. Data were available from twenty published trials.

Published results on tumor control outcomes in terms of local control (LC), regional control (RC), and locoregional control (LRC) and also on survival outcomes in terms of overall survival (OS), distant metastasis-free survival (DMFS), and disease-free survival (DFS) are presented in Table 1. Relevant data are shown according to the patient sample, primary tumor site and stage, treatment intention, median followup, and the percentage of patients that received radiotherapy combined with chemotherapy. The treatment outcomes referred to head and neck cancer patients underwent radiotherapy either with conventional radiotherapy techniques or IMRT. Twenty five trials with available data were analysed in terms of overall survival and locoregional control rate. The mean 3-year overall survival for either IMRT or 2-3D RT was 89.5% (range: 64–100%) and 82.7% (71–88%), respectively. The mean 3-year locoregional control rate 83.6% (range: 70–97%) and 74.4 (range: 61–82%), respectively. The Kruskal-Wallis test revealed a significant (*P* = 0.026) correlation of overall survival with RT technique (IMRT either 2-3DRT), while there was also a significant impact of IMRT technique to locoregional rate (*P* = 0.025). However, according to the bonferroni correction, neither of the above correlations was finally significant. In Table 2, the reported acute and late toxicity rates for mucositis and xerostomia are listed according to the median followup, radiation therapy technique (IMRT or 2-3D RT), and the percentage of patients that received chemotherapy combined with radiotherapy. Few data were available for late mucositis and acute xerostomia concerning the evaluated relevant publications. In the same table, the mean parotid-gland dose is also presented in order to depict the correlation with patient-rated xerostomia. Relevant data with xerostomia grading score and dose deposited at the parotid gland were available in twenty published trials. As shown in Figure 1, the spearman-rho test showed that there was a significant correlation of late xerostomia and mean dose at the parotid gland (rho = 0.5013, *P* < 0.001). According to Figure 2, the mean score of xerostomia for IMRT versus 2-3D RT was 0.31 ± 0.23 and 0.56 ± 0.23, respectively (Mann Whitney, *P* < 0.001). The mean dose deposited in the parotid gland for IMRT versus 2-3DRT was 29.56 ± 5.45 and 50.73 ± 6.79, respectively (Mann Whitney, *P* = 0.016). By analysing thirty five relevant publications with available data with acute mucositis, after comparing the mean values of acute mucositis stratified by IMRT versus 2-3DRT, we found a mean score of 0.71 ± 0.23 versus 0.89 ± 0.07 (Mann-Whitney test, *P* = 0.022), respectively. However, according to bonferroni correction the difference was not significant.

 At last, in Table 3 the range of tumor control and survival rates as well as the range of the acute and late toxicities rates (mucositis and xerostomia) is presented separately for each radiation therapy technique (IMRT versus 2-3D RT).

## 4. Discussion

With the increasing use of IMRT in head and neck carcinomas the improvement of treatment outcomes is the main concern. Several studies in the literature have reported favourable treatment outcomes for patients treated with IMRT technique [8, 9, 24, 25, 28–30, 32, 34, 37, 41, 45]. Comparable rates of LRC are observed in Table 1 among the published studies for different radiotherapy techniques as also comparable survival rates. The comparable LRC and overall survival rates were also confirmed by the descriptive statistical analysis of this study where, beyond a trend in the superiority of IMRT, the differences between the IMRT and 2-3D RT were finally statistically insignificant. However, there are significant variations in tumor control and survival outcomes which are mainly caused by differences in patient sample, tumor stage, and followup among several studies. Chemotherapy also plays a significant role in the variation of clinical results. According to numerous studies, the combination of chemotherapy with radiotherapy improves the efficacy [55–58] at the cost of increased toxicity [55, 56]. In several studies, clinical results were divided according to patients treated with definitive radiotherapy and patients treated with postoperative radiotherapy. In the study of Chao et al. [27], combined surgery and postoperative IMRT lead to improved LRC and DFS compared with definitive IMRT in patients with oropharyngeal carcinoma. Similarly in the study of Studer et al. [48], LC of patients who received definitive IMRT for oral cavity cancer was substantially lower than the LC of patients who received postoperative IMRT.

The published clinical results demonstrate equivalence or noninferiority of IMRT in terms of tumor control or survival in any head and neck site [8–10, 20, 21, 23] while IMRT plays a significant role on the reduction of radiation-induced toxicity [8, 9, 12, 18, 20–23]. According to the published data as shown in Table 3, it seems that IMRT reduces late xerostomia down to 2.3%. According to our descriptive analysis, the mean score of xerostomia was significantly lower in IMRT compared to conventional radiation therapy techniques (Mann Whitney, *P* < 0.001). The prevailing explanation for this inferior toxicity related to IMRT is that the preservation of salivary gland function itself has a protecting effect with regard to radiation-induced oral toxicity and secondary oral infections [59]. Although the data from Table 3 showed that IMRT technique can achieve a reduction of acute mucositis down to 32%, our analysis showed only a trend for the superiority of IMRT.

**Figure 3:**
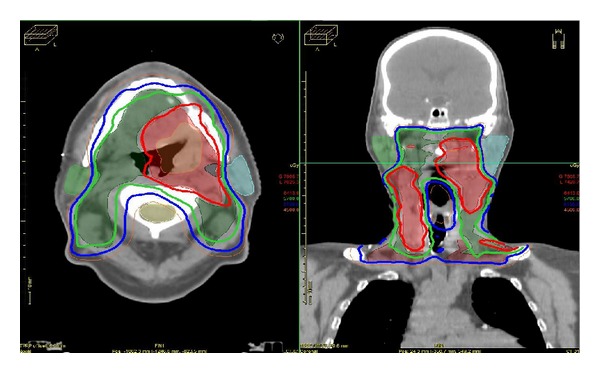
A typical IMRT plan with the parotid sparing technique for a carcinoma of the oral cavity. Three PTVs were contouring: primary site and all relevant lymph nodes as PTV1; primary site and clinical involved lymph nodes as PTV2; primary site only as PTV3. The technique used was integrated boost by means of 30 fractions with 1.8 Gy, 2 Gy, and 2.25 Gy per fraction by PTV1, PTV2, and PTV3, respectively (ONCENTRA, treatment planning). The isodoses shown are the 95% of the prescribed doses per PTV: 45% in orange; 51.3% in blue; 57% in green; 64,13% in red (personal archive).

**Figure 4:**
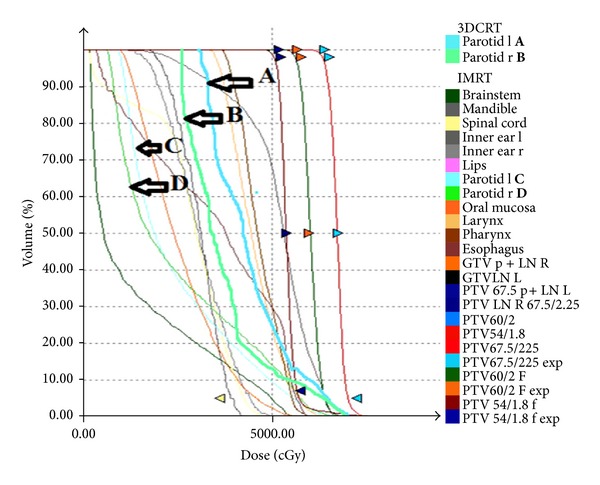
Comparison of the right and left parotid-gland DVHs of the same head and neck cancer patient (tumor site: oral cavity) for IMRT versus 3DCRT (3-dimensional conformal radiotherapy) technique. The arrows show the relevant DVHs for left and right parotid glands. **A** and **B** DVHs for parotids are shifted to the left (**C** and **D**) with IMRT techniques resulting in lower doses in the parotid glands (personal archive).

The mean parotid-gland doses for patients treated with IMRT were significantly lower compared with the mean parotid-gland doses of patients treated with 2-3D RT (Mann Whitney, *P* = 0.016). Furthermore, a significant correlation of late xerostomia and the mean parotid-gland dose was found (spearman test, rho = 0.5013, *P* < 0.001). Numerous studies have also reported significant correlation between the mean parotid dose and salivary flow after RT and the rate of patients suffer from xerostomia [17, 60–66]. A typical IMRT plan with parotid sparing technique for oral cavity carcinoma is shown in Figure 3. Furthermore, the direct comparison of dose volume histogram (DVH) for the right and left parotid gland between IMRT and 3DCRT is presented in Figure 4. The results clearly demonstrate the superiority of IMRT technique in terms of toxicity, mainly due to parotid-gland sparing.

In the literature, comparative studies showed differences regarding tumor control and toxicity profiles among the different radiotherapy techniques (IMRT versus 2-3D RT). In a recent study, Clavel et al. [9] reported superior outcomes (OS, DFS, and LRC) for IMRT patients treated with SIB compared to those treated with conventional radiation therapy techniques for locally advanced oropharyngeal cancer. On the other hand, the majority of the comparative studies demonstrate the equivalence of IMRT with the conventional radiation therapy techniques in regard to tumor control and survival for head and neck cancers [8, 10, 21–23]. In the study of Lee et al. [8], comparable treatment outcomes were observed for IMRT patients and patients treated with 2D conventional radiation therapy technique (2DRT) for locally advanced oropharyngeal carcinoma. Local control and survival appeared slightly superior for IMRT patients compared to 2DRT patients but this difference was statistically insignificant. Similar findings arising for head and neck cancers from the comparison between IMRT and 3DCRT confirm the equivalence in treatment outcomes of IMRT with conventional radiation therapy techniques [10, 18, 21, 23]. Rades et al. [22] compared treatments outcomes among IMRT, 3DCRT, and 2DRT for head and neck cancer patients treated with surgery followed by RT. Locoregional control was similar for the three radiation techniques with IMRT being slightly superior.

As far as late toxicity is concerned, comparative studies report differences between IMRT and conventional RT techniques regarding patient-rated xerostomia [8, 9, 12, 18, 20–23]. Specifically, they demonstrate significantly less xerostomia for head and neck cancer patients treated with IMRT technique than for those treated with conventional radiotherapy techniques. In the studies of Lee et al. [8] and Lambrecht et al. [18], similar results of moderate to severe late xerostomia were observed (12% versus 67% and 23% versus 68% for IMRT and Conv RT, resp.). Clavel et al. [9] reported significant lower xerostomia for IMRT patients compared to those receiveing conventional radiotherapy techniques for locally advanced oropharyngeal carcinoma (IMRT versus conventional RT: 8%, 74%, resp.). Similarly in the study of Rades et al. [22], IMRT is associated with less xerostomia than 2-3D RT for head and neck cancers (17% versus 63% and 73%). Regarding acute toxicity, rates of mucositis for IMRT and conventional RT were reported in several studies [8, 9, 18, 20, 22, 23]. There are studies that demonstrate that patients receiving IMRT had acute toxicity comparable with those receiving 2-3D RT [8, 9, 21–23] while other studies reported that more head and neck cancer patients treated with conventional radiation therapy techniques suffered from acute mucositis compared to those treated with IMRT [18, 20].

In the case that our main concern is the reduction of xerostomia, then IMRT is the appropriate treatment option for head and neck cancer patients. However, when the main aspect is the tumor control or survival, we have to mention the lack of any randomized data to support a recommendation of IMRT over the conventional irradiation beam techniques in any head and neck site. Moreover, in our descriptive analysis, although there was a trend of better treatment outcome in favor of IMRT technique, no significant superiority was noted in terms of either overall survival or locoregional control rate. Definitely, a prospective randomized study comparing the two techniques stands in need. 

## 5. Conclusion

The main conclusion of this study is that IMRT reduces late xerostomia compared with conventional three-dimensional conformal radiotherapy (3D-CRT) and conventional two-dimensional radiotherapy (2DRT). The trend of superiority of IMRT regarding the acute mucositis as well as the overall survival and the locoregional control should be mentioned. However, there is an absence of a clear statistical superiority of IMRT for the various tumor sites as far as tumor control and survival are concerned. A prospective randomized study exploring the potential clinical impact (treatment outcome and radiation-induced toxicity) of IMRT versus 2-3D RT stands in need in order to extract safe conclusions about the definitive superiority of IMRT for tumor control as well as for radiation morbidity. In a parallel way, a meta-analysis with raw data from all relevant publications might also give more definite results in terms of the final potential impact of IMRT for either survival or acute oral mucositis. However, the trend of better treatment outcome in favor of IMRT deriving from our analysis should not be underestimated.

## Figures and Tables

**Table 1:** Published studies with either IMRT or conventional techniques. Data included primary site, stage, intention of RT, concomitant chemotherapy or not, followup, and treatment outcome.

Study	No. of patients	Primary tumor site	TN stage	RT	Intention for RT	Concurrent chemotherapy	Median followup (Range)	Treatment outcome %					
								Regional control (RC)	Local control (LC)	Locoregional control (LRC)	Distant metastases-free survival (DMFS)	Disease-free survival (DFS)	Overall survival (OS)
					no. of pts (%)	no. of pts (%)							
Studer et al. [24]	29	Hypopharynx	T1–T4 N0–N3	IMRT	Def: 90% Post: 10%	YES (86%)	16 mths (4–44 mths)	93% (2yrs)	90% (2yrs)	—	93% (2yrs)	90% (2yrs)	—

Lee et al. [8]	41	Oropharynx	T1–T4 N0–N3 St: III–IVB	IMRT	Def	Yes	31 mths (20–64 mths)	94% (3yrs)	95% (3yrs)	92% (3yrs)	86% (3yrs)	82% (3yrs)	91% (3yrs)
	71	Oropharynx	T1–T4 N0–N3 St: III–IVB	2DRT	Def	Yes	46 mths (3–93 mths)	95% (3yrs)	85% (3yrs)	82% (3yrs)	85% (3yrs)	76% (3yrs)	81% (3yrs)

Garden et al. [25]	51	Oropharynx	Tx, T1-T2 Nx, N0–N3	IMRT	Def	Yes (8%)	45 mths (15–63 mths)	—	96% (2yrs)	93% (2yrs)	—	87% (2yrs)	93% (2yrs)

Daly et al. [26]	107	Oropharynx	T1–T4 N0–N3 St: II–IV	IMRT	Def: 80% Post: 20%	Yes (87%)	29 mths (4–105 mths)	—	—	92% (3yrs)	92% (3yrs)	81% (3yrs)	83% (3yrs)

Chao et al. [27]	74	Oropharynx	T1–T4 N0–N3 St: I–IV	IMRT	Def: 42% Post: 58%	Yes (23%)	33 mths (9–60 mths)	—	—	87% (3yrs)	90% (4yrs)	81% (4yrs)	87% (4yrs)

Huang et al. [28]	71	Oropharynx	T1–T4 N0–N3 St: III-IV	IMRT	Def	Yes	33 mths (3–72 mths)	94% (3yrs)	94% (3yrs)	90% (3yrs)	—	—	83% (3yrs)

Setton et al. [29]	442	Oropharynx	T1–T4 N0–N3 St: I–IV	IMRT	Def: 93% Post: 7%	Yes (88%)	36.8 mths (3–135 mths)	94.4% (3yrs)	94.6% (3yrs)	—	87.5% (3yrs)	—	84.9% (3yrs)

Schoenfeld et al. [30]	64	Oropharynx	T1–T4 N0–N3 St: I–IVB	IMRT	Def	Yes (54%)	36 mths (12–62 mths)		94% (3yrs)	90% (3yrs)	—	RFS: 86% (3yrs)	84% (3yrs)

Clavel et al. [9]	100	Oropharynx	T1–T4 N0–N3 St: III–IVB	IMRT	Def	Yes	42 mths			95.1% (3yrs)		85.3% (3yrs)	92.1% (3yrs)
	149	Oropharynx	T1–T4 N0–N3 St: III–IVB	2-3DRT	Def	YES	42 mths			84.4% (3yrs)		69.3% (3yrs)	75.2% (3yrs)

Sanguineti et al. [31]	50	Oropharynx	Tx, T1–T4 N0–N3	IMRT	Def	No	32.6 mths (12.1–58.6 mths)	85% (3yrs)	94% (3yrs)				

De Arruda et al. [32]	50	Oropharynx	T1–T4 N0–N3 St: I–IV	IMRT	Def: 96% Post: 4%	Yes (86%)	18 mths (8.4–76 mths)	88% (2yrs)	98% (2yrs)		84% (2yrs)		98% (2yrs)

Fang et al. [10]	113	Nasopharynx	T1–T4 N0–N3 St: I–IV	IMRT	Def	Yes (57.3%)	40 mths (5–57 mths)	—	—	84% (3yrs)	83% (3yrs)	—	85% (3yrs)
	93	Nasopharynx	T1–T4 N0–N3 St: I–IV	3D-CRT	Def	Yes (54.8%)	46 mths (10–59 mths)	—	—	84.8% (3yrs)	76.7% (3yrs)	—	81.7% (3yrs)

Wong et al. [33]	175	Nasopharynx	T1–T4 N0–N3 St: I–IVB	IMRT	Def	Yes (70%)	34 mths (9–50 mths)	93.3% (3yrs)	93.6% (3yrs)		86.6% (3yrs)		87.2% (3yrs)

Kwong et al. [34]	33	Nasopharynx	T1–T3 N0-N1	IMRT	Def	No	24 mths (11–42 mths)	93% (3yrs)	100% (3yrs)	—	100% (3yrs)	—	100% (3yrs)

Wolden et al. [35]	74	Nasopharynx	T1–T4 N0–N3 St: I–IVB	IMRT	Def	Yes (93%)	35 mths (3–74 mths)	93% (3yrs)	91% (3yrs)	—	78% (3yrs)	67% (3yrs)	83% (3yrs)

Kam et al. [36]	63	Nasopharynx	T1–T4 N0–N3 St: I–IV	IMRT	Def	Yes (30%)	29 mths (8–45 mths)	98% (3yrs)	92% (3yrs)	—	79% (3yrs)	—	90% (3yrs)

Tham et al. [37]	107	Nasopharynx	T1–T2 N0–N1 St: IIB	IMRT	Def	Yes (7%)	39 mths (7–77 mths)	98% (3yrs)	96.5% (3yrs)		94.8% (3yrs)	90.7% (3yrs)	95.8% (3yrs)

Lee et al. [38]	68	Nasopharynx	T1–T4 N0–N3 St: I–IVB	IMRT	Def	Yes (65%)	31 mths (6–55 mths)	90.8% (2yrs)	92.6% (2yrs)	89.3% (2yrs)	84.7% (2yrs)	72.7% (2yrs)	80.2% (2yrs)

Tham et al. [39]	195	Nasopharynx	T1–T4 N0–N3 St: I–IVB	IMRT	Def	Yes (57%)	36.5 mths		89.6% (3yrs)		89.2% (3yrs)	79% (3yrs)	94.3% (3yrs)

Lee et al. [40]	67	Nasopharynx	T1–T4 N0–N3 St: I–IV	IMRT	Def	Yes (75%)	31 mths (7–72 mths)	98% (4yrs)	97% (4yrs)	—	66% (4yrs)	—	88% (4yrs)

Liu et al. [41]	19	Nasopharynx	T1–T4 N0–N3 St: I–IV	IMRT	Def	Yes (58%)	13.0 mths (8–18 mths)	—	—	100% (13 mths)			

Luo et al. [42]	58	Nasopharynx	T1–T4 N0–N3 St: I-II	3D-CRT	Def	No	58 mths (25–92 mths)	—	—	93% (5yrs)	98% (5yrs)	91% (5yrs)	95% (5yrs)

Wang et al. [43]	300	Nasopharynx	T1–T4 N0–N3 St: IIB	IMRT	Def	Yes (83%)	47.1 mths (11–68 mths)	95.1% (4yrs)	94% (4yrs)	—	85% (4yrs)	—	86.1% (4yrs)

Sultanem et al. [44]	35	Nasopharynx	T1–T4 N0–N3 St: I–IVB	IMRT	Def	Yes (91%)	21.8 mths (5–49 mths)			100% (4yrs)	57% (4yrs)	57% (4yrs)	94% (4yrs)

Su et al. [45]	198	Nasopharynx	T1-T2 N0-N1 St: I–IIB	IMRT	Def	No	50.9 mths (12–104 mths)		97.7% (5yrs)		97.8% (5yrs)	97.3% (5yrs)	

Kim et al. [46]	21	Nasopharynx	T2–T4 N0–N2 St: III-IV	3D-CRT	Def	No	48 mths					85% (5yrs)	61% (5yrs)

Al-Mamgani et al. [47]	170	Larynx	T3 N0–N3	IMRT + 3D-CRT	Def	Yes (28.3%)	32 mths (7–172 mths)	—	73% (3yrs)	70% (3yrs)		64% (3yrs)	61% (3yrs)

Studer et al. [48]	58	Oral cavity	*Def* T2–T4 N0–N3 St: IVA-B *Post* T1–T4 N0–N2c St: II–IVA	IMRT	Def: 52% Post: 48%	Yes (78%)	Def: 19 mths Post: 12 mths		Def: 43% (2yrs) Post: 92% (2yrs)	—	—	Def: 40% (2yrs) Post: 87% (2yrs)	Def: 30% (2yrs) Post: 83% (2yrs)

Chen et al. [21]	22	Oral cavity	T1–T4 N0–N2c St: III-IV	IMRT	Post	Yes (9%)	44 mths					64% (3yrs)	67% (3yrs)
	27	Oral cavity	T1–T4 N0–N2c St: III-IV	3D-CRT	Post	Yes (2%)	44 mths					66% (3yrs)	77% (3yrs)

Yao et al. [49]	55	Oral cavity	Tx–T4 N0–N2c St: I–IV	IMRT	Def: 9% Post: 91%	Yes Def: 6% Post: 4%	23.9 mths (9.3–59.3 mths)		85% (3yrs)	82% (3yrs)	89% (3yrs)	74% (3yrs)*	68% (3yrs)

Lee et al. [50]	31	Various	T1–T4 N0–N2 St: I–IVB	IMRT	Def	Yes (65%)	26 mths (17–58 mths)		86% (2yrs)	94% (2yrs)	92% (2yrs)		63% (2yrs)

Van Gestel et al. [51]	78	Various	Tx, T1–T4 Nx, N0–N3 St: I–IVB	IMRT	Def: 62% Post: 38%	Yes Def: 63% Post: 23%	18.7 mths (0.13–51.7 mths)			Def: 66.8% (3yrs) Post: 82.2% (3yrs)		Def: 42.6% (3yrs) Post: 82.2% (3yrs)	Def: 60.3% (3yrs) Post: 85.9% (3yrs)

Peponi et al. [52]	82	Various	St: I–IV	IMRT	Def: 77% Post: 23%	Yes (85%)	55 mths		78% (3yrs)				80% (3yrs)

Gupta et al. [23]	28	Various	T1–T3 N0–N2b St: I–IV	3D-CRT	Def	Yes	40 mths (26–50 mths)			88.2% (3yrs)			70.6% (3yrs)
	32	Various	T1–T3 N0–N2b St: I–IV	IMRT	Def	Yes (90%)	40 mths (26–50 mths)			88.2% (3yrs)			68% (3yrs)

Lambrecht et al. [18]	135	Various	T1–T4 N0–N3 St: III-IV	3D-CRT	Def	Yes (80%)	68 mths (37.2–104 mths)			71% (3yrs)			61% (3yrs)
	110	Various	T1–T4 N0–N3 St: III-IV	IMRT	Def	Yes (81%)	35 mths (4.7–63.5 mths)			70% (3yrs)			64% (3yrs)

Toledano et al. [53]	208	Various	St: I–IV	IMRT	Def: 55% Post: 45%	Yes (37.5%)	25.3 mths (0.4–72 mths)	—	—	86% (2yrs)	92.7% (2yrs)	80% (2yrs)	86.7% (2yrs)

Rades et al. [22]	104	Various	T0–T4 N0–N3 St: I–IV	2DRT	Post	Yes (8%)			78% (2yrs)		79% (2yrs)		74% (2yrs)
	26	Various	T0–T4 N0–N3 St: I–IV	3D-CRT	Post	Yes (23%)			79% (2yrs)		83% (2yrs)		80% (2yrs)
	18	Various	T0–T4 N0–N3 St: I–IV	IMRT	Post	Yes (6%)			89% (2yrs)		80% (2yrs)		86% (2yrs)

Yao et al. [54]	150	Various	T0–T4 N0–N3 St: I–IV	IMRT	Def: 66% Post: 34%	Yes (45%)	18 mths (2–60 mths)		94% (2yrs)	92% (2yrs)	87% (2yrs)		85% (2yrs)

**Table 2:** Published studies with either IMRT or conventional techniques. Data included concomitant chemotherapy or not, follow-up, and radiation-induced toxicity.

Study	Radiation treatment	Concurrent chemotherapy No. of patients (%)	Median followup (range)	Toxicity (≥GrII)				Mean parotid- gland dose
				Mucositis		Xerostomia	
				Acute	Late	Acute	Late
Studer et al. [24]^†^	IMRT	Yes (86%)	16 mths (4–44 mths)	65%	—	—	—	
Lee et al. [8]	IMRT	Yes	31 mths (20–64 mths)	66%	—	66%	12%	
	2DRT	Yes	46 mths (3–93 mths)	72%		65%	67%	
Garden et al. [25]	IMRT	Yes (8%)	45 mths (15–63 mths)	—	—	—	—	23.9 Gy
Daly et al. [26]^†^	IMRT	Yes (87%)	29 mths (4–105 mths)	93%	—	—	—	33.2 Gy
Chao et al. [27]^†^	IMRT	Yes (23%)	33 mths (9–60 mths)	86%	—	—	12%	18.6 Gy
Huang et al. [28]	IMRT	Yes	33 mths (3–72 mths)	92%	—	—	34%	25.5 Gy
Setton et al. [29]^†^	IMRT	Yes (88%)	36.8 mths (3–135 mths)	68%	—	28%	29%	25.8 Gy
Kwong et al. [34]	IMRT	No	24 mths (11–42 mths)	82%	—	—	40% (1yrs) 15% (2yrs)	38.8 Gy
Wolden et al. [35]	IMRT	Yes (93%)	35 mths (3–74 mths)				32% (1yrs)	35.2 Gy
Kam et al. [36]	IMRT	Yes (30%)	29 mths (8–45 mths)	92%		75%	23% (2yrs)	39 Gy
							16.7% (2yrs)	31 Gy
Lee et al. [38]	IMRT	Yes (65%)	31 mths (6–55 mths)	—	22%	—	33%	
Lee et al. [40]	IMRT	Yes (75%)	31 mths (7–72 mths)	94%	—		58%	
Liu et al. [41]	IMRT	Yes (58%)	13.0 mths (8–18 mths)	—	79%	—	53%	37.8 Gy
Luo et al. [42]	3D-CRT	No	58 mths (25–92 mths)	74%	12%		12%	52.8 Gy
Al-Mamgani et al. [47]	IMRT + 3D-CRT	Yes(28.3%)	32 mths (7–172 mths)				14.1%	23.6 Gy
Lee et al. [50]	IMRT	Yes (65%)	26 mths (17–58 mths)	48%			3.2%	26 Gy
Van Gestel et al. [51]^†^	IMRT	Yes Def: 63% Post: 23%	18.7 mths (0.13–51.7 mths)	100%			44%	
Peponi et al. [52]^†^	IMRT	Yes (85%)	55 mths				Obj: 7.3 % Sub: 3.6%	
Gupta et al. [23]	3D-CRT	Yes	40 mths (26–50 mths)	93%				53 Gy
	IMRT	Yes (90%)	40 mths (26–50 mths)	77%				34.3 Gy
Lambrecht et al. [18]	3D-CRT	Yes (80%)	68 mths (37.2–104 mths)	44%			68%	53 Gy
	IMRT	Yes (81%)	35 mths (4.7–63.5 mths)	32%			23%	34 Gy
Toledano et al. [53]^†^	IMRT	Yes (37.5%)	25.3 mths (0.4–72 mths)	~73%	—	—	~58%	
Clavel et al. [9]	IMRT	Yes	42 mths	75%			8% (2yrs)	
	2-3DRT	Yes	42 mths	77%			74% (2yrs)	
Vergeer et al. [20]^†^	IMRT	Yes (43%)	—				32% (6 mths)	27 Gy
	3D-CRT	Yes (35%)	—				56% (6 mths)	43 Gy
Chen et al. [21]*	IMRT	Yes (9%)	44 mths	87%			36%	
	3D-CRT	Yes (2%)	44 mths	89%			82%	
Nutting et al. [12]^†^	IMRT	Yes (43%)	44 mths	93%		71%	69%	36.5 Gy
	3D-CRT	Yes (40%)	44 mths	94%		91%	76%	61 Gy
Wong et al. [33]	IMRT	Yes (70%)	34 mths (9–50 mths)	67.4%			2.3%	30 Gy
Sultanem et al. [44]	IMRT	Yes (91%)	21.8 mths (5–49 mths)	97%			28%	
Su et al. [45]	IMRT	No	50.9 mths (12–104 mths)	73%		36%	15.4% (1yrs) 9.0% (2yrs)	31 Gy
Wang et al. [43]	IMRT	Yes (83%)	47.1 mths (11–68 mths)	33.3%		4.7%	12.3% (2yrs)	27.6 Gy
Rades et al. [22]*	2DRT	Yes (8%)		~90%			73%	
	3D-CRT	Yes (23%)		~90%			63%	
	IMRT	Yes (6%)		~90%			17%	
Tham et al. [39]	IMRT	Yes	36.5 mths	29% (Gr3)		3% (Gr3)		
	IMRT	No	36.5 mths	20% (Gr3)				
Kim et al. [46]	3D-CRT	No	48 mths	57%			19%	
De Arruda et al. [32]^†^	IMRT	Yes (86%)	18 mths (8.4–76 mths)	92%		60%	33%	26.5 Gy

*Postoperative RT; ^†^definitive and postoperative RT; all the rest: definitive RT.

**Table 3:** Synoptic table with ranges of treatment outcome and toxicity between IMRT versus conventional techniques.

RT	Treatment outcome					
	RC	LC	LRC	DMFS	DFS	OS
IMRT	85%–98%	78%–100%	70%–100%	57%–100%	57%–97.3%	63%–100%
2DRT-3DCRT	95%*	78%–85%	71%–93%	76.7%–98%	66%–91%	61%–95%

	Acute toxicity			Late toxicity		
	Mucositis	Xerostomia		Mucositis	Xerostomia	

IMRT	32%–100%	4.7%–75%		22%–79%	2.3%–69%	
2DRT-3DCRT	44%–94%	65%–91%		12%*	12%–82%	

*Available data from one study only.
